# Patient-reported outcomes after initial conservative fracture treatment in primary healthcare – a survey study

**DOI:** 10.1186/s12875-022-01799-4

**Published:** 2022-07-30

**Authors:** Stein Vabo, Knut Steen, Christina Brudvik, Steinar Hunskaar, Tone Morken

**Affiliations:** 1 National Centre for Emergency Primary Health Care, NORCE Norwegian Research Centre, Box 22 Nygårdstangen, 5838 Bergen, Norway; 2 Vennesla Health Care Center, Sentrumsvegen 41, 4700 Vennesla, Norway; 3 Department of Global Public Health and Primary Care, University of Bergen, P.O. Box 7800, 5020 Bergen, Norway; 4 Department of Clinical Medicine, University of Bergen, P.O. Box 7800, 5020 Bergen, Norway

**Keywords:** Conservative fracture treatment, General practice, Primary healthcare, Teleradiology, Diagnosis

## Abstract

**Background:**

Primary healthcare in Norway has first-line responsibility for medical emergencies, including traumas and fractures. Normally, patients with suspected fractures are referred to specialist care. However, some municipalities have X-ray facilities and handle minor fractures locally. We investigated patient-reported outcome measures after initial treatment of radiologically diagnosed fractures of the wrist, collarbone, and ankle at a primary healthcare centre in a rural municipality with a large ski resort. The patients’ general satisfaction with the treatment was also investigated.

**Methods:**

Validated questionnaires were sent to patients with fractures of the wrist or collarbone (Quick DASH—Disability of Arm, Shoulder and Hand) or the ankle (FAOS -The Foot and Ankle Outcome Score). Patients with wrist and collarbone fractures also answered the Quality-of-life questions that are a subscale of the FAOS questionnaire for ankle fractures. Patient satisfaction was measured for all fracture groups. The Quick DASH scale ranges from 0 (no disability at all) to 100 (great disability), while for FAOS a score of 100 indicates no symptoms and 0 indicates extreme disabilities.

**Results:**

A total of 148 of 238 patients answered the questionnaire (62% response rate). Patients with distal radius fractures had a mean Quick DASH score of 5.1 (median 0, range 0–77), and scores were significantly lower for males (*p* = 0.013) and increased with age (*p* = 0.024). Patients with collarbone fractures had a mean Quick DASH score of 2.1 (median 0, range 0–32) with no significant age or gender differences. Patients with ankle fractures had the following mean subscale-scores: Pain, 93.8; Symptoms, 71.4; Activities of daily living, 97.4; Sport, 90.0; and Quality of life, 92.1. The scores did not differ significantly by specialization of the physician. A total of 88% of the patients were highly or very highly satisfied with the handling of their fracture.

**Conclusions:**

The patients reported low rates of functional disability and high rates of satisfaction after initial radiological diagnosis and treatment of their fracture at the primary healthcare centre. Specialisation of the treating physician was not associated with the outcome in any of the fracture types.

## Background

Primary healthcare in Norway has first-line responsibility for all medical emergencies, including traumas such as fractures. Normally, patients with suspected fractures are referred to specialist healthcare for treatment because most primary healthcare clinics do not have radiological services. However, some municipalities in Norway and elsewhere in Europe have invested in X-ray facilities and treat fractures in primary healthcare [1, 2].

In a previous study we found that almost 80% of all patients diagnosed with fractures at a primary care centre in Norway were initially treated with casts, braces, or slings at the primary care level [2]. The other 20% were sent to hospital for surgical treatment of the fractures.

Research on patient reported outcome measures (PROMs) after initial conservative treatment of fractures in primary care is scarce. Most studies of outcomes after wrist, collarbone, or ankle fractures are based on PROM data from patients treated in hospitals [3–9].

Conservative treatment, including a reduction of the displacement, a stabilising cast, and follow-ups to control the maintanance of the fracture reduction still have an important place in the treatment of non-displaced and slightly displaced distal radius, collarbone, and ankle fractures [3, 4]. Despite this, only a few studies have been published on fracture diagnostics or treatment in primary healthcare, including the work of a Dutch research group that has addressed this topic in three different publications [1, 10, 11]. They have investigated fracture diagnostics, unnecessary travel and treatment, and the situation before and after the introduction of teleradiology in a remote general practice [1], a cost–benefit analysis in this setting [10], and patient satisfaction with a teleradiology service in general practice [11]. However, none of the identified studies on fracture treatment in primary healthcare included PROM data after conservative fracture treatment.

Due to the scarcity of knowledge regarding outcomes after fracture treatment in primary healthcare, the aims of this study were to assess: 1) PROM data after initial treatment of radiologically diagnosed fractures of the wrist, collarbone, or ankle at a primary healthcare centre, 2) differences in outcome scores by patients’ age and gender and by specialisation of the treating physician, and 3) patients’ general satisfaction with the fracture treatment given at the primary healthcare centre.

## Methods

### Setting

The study was performed among patients in Bykle municipality, Norway. In the municipality, there is a remote rural ski resort with a three hours’ drive to the nearest hospital. Due to a high volume of fractures and its geographic remoteness, the primary care health centre invested in X-ray equipment in 2004, with the possibility to transfer the X-ray images electronically to the nearest hospital. When a patient is likely to have a fracture, radiological diagnostics are performed at the local primary healthcare centre. The X-ray images are digitalised and instantly transferred for further assessment by a radiologist or an orthopaedic surgeon at the regional hospital both before and after initial treatment. If necessary, the physician on call at the healthcare centre can ask for an almost instant radiological assessment by a consultant. The X-ray examinations and fracture treatments are based on recommended and updated knowledge and are performed in accordance with the radiological and orthopaedic procedures at the regional hospital [12]. The physician on call at the primary healthcare centre provides the initial fracture treatment, but most cases are followed-up by orthopaedic surgeons at the hospital or by specialist services after referral from the primary healthcare centre.

Normally the primary healthcare centre is staffed by general practitioners, but during the skiing season, when the population rises tenfold, the medical centre is also served by extra physicians with a large variety of medical backgrounds. Clinical experience for the physicians on call varies from interns in general practice, with one-year postgraduate practice, to specialists in general practice with decades of clinical experience. Other medical specialities vary from orthopaedic surgery to general surgery, internal medicine, neurology, and gynaecology, and most of them are recruited from hospitals in Sweden and Denmark.

The orthopedic surgery group consisted of four physicians over the five years. The non-orthopedic group consisted of 24 physicians. Some interns in general practice, all with recent experience from orthopedic treatment, some specialists in general practice, and a mixed group of physicians who were general practitioners undergoing training or more experienced branch specialists with experience from Norwegian general practice.

Basic interpretation of x-rays and basic initial fracture treatment are included in the undergraduate medical education in all Scandinavian countries, but all non-orthopaedic physicians still receive updated training at the primary healthcare centre before starting their duties.

### Sample and data collection

All Norwegian and Danish patients initially diagnosed and initially treated for fracture of the radius, collarbone, or ankle during the five-year period from 2010 and through 2014 were eligible for inclusion. The first author (SV) reviewed all medical records at Bykle primary healthcare centre to identify the patients. The following variables were registered: fracture type and localisation and specialization of the physician who treated the patient at the primary healthcare centre. The physicians were categorised into two types: 1) specialist in orthopaedics and 2) non-orthopaedics, including interns, specialists in general practice, and other specialists.

**Fig. 1:**
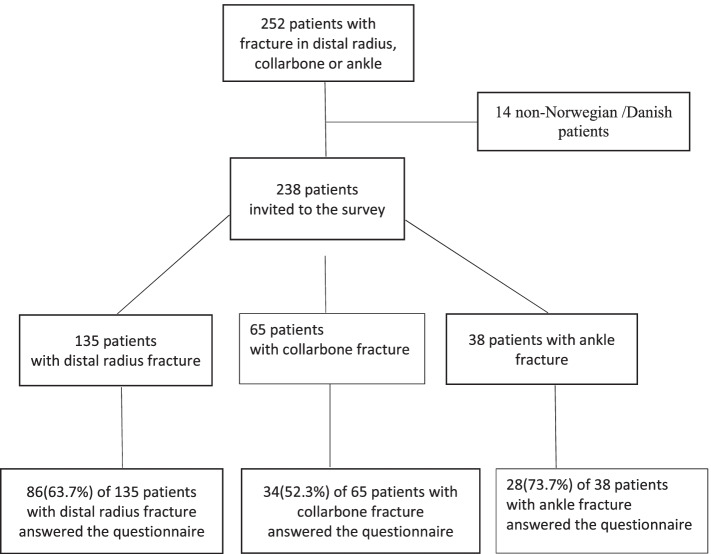
Flowchart of the included patients

We chose patients with X-ray-verified fractures of the wrist, collarbone, or ankle in the survey study (Fig. 1) because these fractures are the most common fractures and constitute a large proportion of patients with fractures in this specific primary healthcare setting [2, 13].

### The questionnaire

In 2015, a questionnaire and information about the study was sent to all eligible patients. They also received written information about the study. The questionnaire was written in Norwegian and sent to both Norwegian and Danish patients as written Norwegian is understandable to Danes. For patients below 16 years of age, the closest relative was asked to fill in the questionnaire. Reminders were sent to non-responders after one and two months.

The questionnaire had three parts:

1. General information:
2. age and gender of the patient
3. type of injury (categorized into skiing/not skiing-related)
4. surgical treatment of the fracture (yes/no)
5. previous injury in the same extremity with need of treatment by a physician (yes/no).

2. Patient satisfaction

- “In total, was the help from the emergency primary healthcare satisfactory?”
- “Do you think that you in some ways were incorrectly treated?”
- “In total, how did you experience the help you received from the emergency primary healthcare?”
  The answers were graded from 0 to 4. For the two first questions, the alternatives were: Not at all, to a low degree, to some degree, to a high degree, or to very high degree. For the last question, the alternatives were: Much worse than expected, worse than expected, as expected, better than expected, or much better than expected.

3. PROMs

The questionnaire included validated PROMs adapted to the three different types of fracture. The Quick DASH outcome measure was sent to patients with radius or clavicular fractures. The FAOS was sent to patients with ankle fractures.

- Quick DASH
  The Quick DASH questionnaire evaluates disability and symptoms of the upper extremities [14], and it is often used in orthopaedics to differentiate small and large changes of disability over time after surgery [6]. It is a short version of the original DASH questionnaire (30 questions), and the quick version with 11 questions has been shown to have satisfactory validity and reliability to reflect everyday functional outcome after fracture injury [14]. Each question on the Quick DASH questionnaire is rated on a Likert scale from 0 to 4. The average score from all 11 questions is multiplied by 25, and this gives a Quick DASH score ranging from 0 (no disability at all) to 100 (greatest possible disability). At least 9 of the 11 questions on the Quick DASH must be answered to calculate a total score. The Quick DASH has been translated into Norwegian and validated [15], and normative values have also been collected in the general population in Norway [16].
- FAOS
  The FAOS is a patient-reported questionnaire with 42 items assessing functional outcome after a variety of foot and ankle-related problems during the previous week. It was primarily developed in Sweden and has been translated to English and other languages and is widely used. The validity and reliability appear to be satisfactory [17], although the Norwegian version of the FAOS has not yet been validated in studies. The FAOS consists of five subscales: Pain, Other symptoms, Activities of daily living (ADL), Function in sport and recreation (Sport/Rec), and Foot and ankle-related quality of life (QOL). Standardised answer options are given, and each question gets a score on a Likert scale from 0 to 4. A score is calculated for each subscale and transformed to a scale where 100 indicates no symptoms at all and 0 indicates extreme disability.
- QOL

The four questions in the QOL subscale in the FAOS questionnaire were included in the questionnaire for the patients with distal radius fracture and patients with collarbone fracture. Standardised answer options were given, and each question could get a score from 0 to 4. A summary score was calculated and transformed to a scale where 100 indicated the highest QOL and 0 indicated the lowest QOL.

### Statistics

Descriptive statistics were used to find absolute numbers, percentages, means, median and range. Comparisons of proportions were tested with chi-square tests, and differences in age, Quick DASH score, and FAOS score between groups were tested with the Mann–Whitney U-tests. Correlations between age and Quick DASH score/FAOS score were tested with Pearson’s correlation coefficient. Statistical significance was set at *p* < 0.05. The software program IBM® SPSS® Statistics version 25 was used for statistical analyses.

## Results

We identified 238 eligible patients, including 135 patients with distal radius fractures, 65 with collarbone fractures, and 38 with ankle fractures. A total of 148 of the 238 patients answered the questionnaire a response rate of 62.2%. The response rate varied depending on type of fracture, and 86 (63.7%) of the patients with distal radius fracture, 34 (52.3%) of the patients with collarbone fracture, and 28 (73.7%) of the patients with ankle fracture answered the questionnaire.

A total of 28 (18.9%) of the questionnaires were completed by the patient’s closest relative, and 82 (55.4%) of the patients were men (Table 1). Responders were not significantly older compared to non-responders. Significantly more women (66 (77.6%) out of 85) than men (82 (53.6%) out of 153) answered the questionnaire, and the mean age at time of injury was 29.3 years. A total of 102 patients (68.9%) reported that the injury occurred when skiing, 27 patients (18.4%) reported that they had received surgical treatment for the fracture, and 13 patients (9%) had previously experienced an injury in the same extremity (Table 1).

**Table 1:** Information about patients and trauma

	All patients (*n* = 148)		Distal radius (*n* = 86)		Collarbone (*n* = 34)		Ankle (*n* = 28)	
	Median (range)	Mean	Median (range)	Mean	Median (range)	Mean	Median (range)	Mean
Age at time of injury	17 (3–75)	29.3	15 (3–75)	26.5	14.5 (4–60)	23.8	50 (6–72)	44.6
	n	%	n	%	n	%	n	%
Gender								
Male	82	(55.4)	42	(48.8)	28	(82.4)	12	(42.9)
Female	66	(44.6)	44	(51.2)	6	(17.6)	16	(57.1)
Type of injury								
Ski	102	(68.9)	54	(63.5)	29	(87.9)	19	(67.9)
Not ski	46	(30.1)	32	(36.5)	5	(12.1)	9	(32.1)
Surgical treatment								
Yes	27	(18.4)	14	(16.3)	4	(11.8)	9	(32.1)
No	120	(81.6)	72	(83.7)	30	(88.2)	19	(67.9)
Previous injury of same extremity								
Yes	13	(9.0)	9	(10.5)	3	(9.1)	1	(9.0)
No	135	(91.0)	77	(89.5)	31	(90.9)	27	(91.0)

### The treating physicians

A total of 26 patients (17.6%) were initially diagnosed and treated by a specialist in orthopaedics at the primary healthcare centre (16 of whom had distal radius fractures, 6 had collarbone fractures, and 5 had ankle fractures). The other 122 patients (82.4%) were diagnosed and treated by non-orthopaedic doctors. This group consisted of 24 physicians, interns did 35.8% of the diagnosis/treatments, specialists in general practice 22.3% and other physicians 24.3%.

A total of 120 (81.6%) of the respondents were treated locally by the primary healthcare service, and 27 patients (18.4%) were sent to hospital for surgery (14 wrist, 4 collarbones, and 9 ankle fractures).

### Distal radius fractures

Of the 86 respondents with distal radius fractures, 44 (51.2%) were female, and the mean age at time of injury was 26.5 years. The female patients were significantly older (33.9 years) than the male patients (18.7 years) (*p* < 0.001). Fifty-four patients (63.5%) sustained the fracture during skiing, and 14 (16.5%) of the patients had received surgical treatment. The mean Quick DASH score among the patients with distal radius fracture was 5.1 (median 0, range 0–77), and the score was significantly lower for male patients (mean 2.0, median 0, range 0–16) than for female patients (mean 8.1, median 0, range 0–77). Table 2 shows the Quick DASH scores by age categories among male and female patients with distal radius fracture. The Quick DASH score increased with increasing age. There were no significant differences in Quick DASH scores between the patients treated by specialists in orthopaedics and non-orthopaedics.

**Table 2:** Quick DASH scores among patients with distal radius fracture or collarbone fracture (*n* = 120)

		Distal radius fracture			Collarbone fracture		
	Age	n	Median (range)	Mean	n	Median (range)	Mean
Female							
	0–19	19	0 (0–77)	6.0	4	0.0 (0)	0.0
	20–29	2	12.5 (0–25)	12.5	0		
	30–39	3	3.0 (0–9)	3.0	0		
	40–49	6	17.0 (0–48)	17.0	1	0.0 (0)	0.0
	50–59	4	14.8 (0–16)	11.4	1	0.0 (0)	0.0
	60–69	7	0 (0–9)	2.3	0		
	70 +	2	18.2 (16–20)	18.2	0		
Male							
	0–19	36	0 (0–14)	1.5	19	0 (0–32)	3.0
	20–29	1	0 (0)	0.0	0		
	30–39	0			0		
	40–49	0			4	0 (0–2)	0.6
	50–59	2	8.0 (0–16)	8.0	3	4,5 (0–7)	3.8
	60–69	2	5.7 (0–11)	5.7	1	0.0 (0)	0
	70 +	0			0		

### Collarbone fractures

Twenty-eight (82.4%) of the patients were male, and the mean age was 23.8 years. There was no age difference between female (24.8 years) and male (23.6 years) patients. Four patients had received surgical treatment (11.8%). Twenty-nine patients (87.9%) stated that the reason for the fracture was a skiing injury. The mean Quick DASH score among the 34 patients with collarbone fracture was 2.1 (median 0, range 0–32). The difference in score between male patients (mean 2.6, median 0, range 0–32) and female patients (mean 0, median 0) was not significant (*p* = 0.348) (Table 2). There were no significant correlations between the Quick DASH score and age (*p* = 0.173), and there were no significant differences in Quick DASH scores between the patients treated by specialists in orthopaedics and by other types of physicians (*p* = 0.874).

### Ankle fracture

Sixteen (57.1%) of the patients were female, and the mean age was 44.6 years. There was no significant age difference between female (mean 50.9 years, median 53, range 23–68) and male (mean 36.2 years, median 32, range 6–72) patients (p = 0.058). Nine patients (32.1%) had received surgical treatment, and 19 patients (67.9%) stated that the reason for the fracture was a skiing injury. Table 3 shows the FAOS scores among patients with an ankle fracture. The subscale “symptoms” had the lowest score with a mean score of 68.1 for all patients, and a mean score of 66.3 for female patients.

**Table 3:** Foot and ankle score (FAOS) among patients with ankle fracture

	All (*n* = 28)		Male (*n* = 12)		Female (*n* = 16)		
	Median (range)	Mean	Median (range)	Mean	Median (range)	Mean	*P*-value
Pain	100.0 (67–100)	93.8	95.8 (67–100)	90.5	100.0 (81–100)	96.4	0.152
Symptoms	71.4 (54–86)	68.1	71.4 (61–82)	70.5	68.0 (54–86)	66.3	0.184
Activities of daily living	100.0 (75–100)	97.4	100.0 (75–100)	95.6	100.0 (90–100)	98.8	0.187
Sport	95.0 (65–100)	90.0	95.0 (65–100)	88.3	90.0 (70–100)	91.3	0.499
Quality of life	100.0 (38–100)	87.7	93.8 (38–100)	82.3	100.0 (56–100)	92.1	0.166

^*^ One patient did not answer questions about Activities of daily living, Sport, or Quality of life

We found significant correlation between ADL-score and age (*p* = 0.015). There was no significant correlation between age and each of the other FAOS subscale scores: pain, symptoms, sports or quality of life.

### QOL

The QOL score for wrist fracture was 92.1 (*n* = 85), for collar bone fracture 95.3 (*n* = 33), and for ankle fracture 92.1 (*n* = 28) (Table 4). For patients with ankle fracture, there was no significant gender difference (*p* = 0.166). For patients with distal radius fracture, male patients reported significantly higher QOL than female patients (*p* = 0.014). For patients with collarbone fracture, the mean QOL was 95.3. Female patients reported significantly higher QOL than male patients (*p* = 0.006).

**Table 4:** Quality of life (QOL) among patients with distal radius, collarbone, and ankle fractures

	All patients		Male patients		Female patients		
	Median (range)	Mean	Median (range)	Mean	Median (range)	Mean	*p*-value
Distal radius fracture *(n* = 85)	100.0 (37–100)	92.1	100.0 (56–100)	96.1	100.0 (37–100)	88.2	0.014
Collarbone fracture (*n* = 33)	100.0 (69–100)	95.3	100.0 (69–100)	94.2	100.0 (100–100)	100.0	0.006
Ankle fracture *(n* = 27)	100.0 (38–100)	87.7	93.8 (38–100)	82.3	100.0 (56–100)	92.1	0.166

### Satisfaction with the emergency primary healthcare service

Table 5 shows that 88.4% of the patients were satisfied to a high or very high degree with the fracture management that was provided at the primary healthcare centre. Fourteen patients (9.5%) reported that the help from the emergency primary healthcare was worse than expected, and 9 patients (6.1%) felt that they received improper fracture treatment to a high or very high degree.

**Table 5:** Reported satisfaction with the emergency primary healthcare service

	n	%	n	%	n	%
	Not at all/to a less degree		To some degree		To a high/very high degree	
In total, was the help from emergency primary healthcare satisfactory? (*n* = 146)	8	(5.5)	9	(6.2)	129	(88.4)
Do you think that you in some ways were maltreated? (*n* = 148)	131	(88.5)	8	(5.4)	9	(6.1)
	Worse/much worse than expected		As expected		Better/much better than expected	
In total, how did you experience the help from emergency primary healthcare? (*n* = 147)	14	(9.5)	48	(32.7)	85	(57.8)

## Discussion

### General findings

To our knowledge, this is the first study to address PROM data and other measures of outcome after primary diagnostics and initial conservative treatment of fractures in general practice. Nearly 90% of the patients reported a high or very high degree of satisfaction with the initial treatment of fractures performed at the primary healthcare centre. Satisfaction was paralleled by the results from the PROM data for the different fractures. In general, the patients reported low rates of disability after management of fractures of the wrist, collarbone, or ankle.

The mean Quick DASH scores for both wrist fractures and collarbone fractures were low, which indicates good functional results and little pain. FAOS subscale scores for the ankle were in the upper third of the scale and indicate a low degree of functional problems and pain. In the metanalysis from Larsen et al. [8], including eight studies with PROMs, the main conclusion was that the best available current evidence supports that clinicians outside the hospital can manage the treatment of both stable and unstable non-displaced ankle fractures with conservative means. In this selected patient group, the short-term results are equal to operative treatment. This study gives support to the approach we used in the initial handling of ankle fractures in our rural setting. Based on X-rays and clinical picture with decision-support by radiologist and an orthopaedic surgeon at the collaborating hospital, we singled out patients in need of surgery at the hospital and which patients could be treated conservatively at the primary healthcare centre.

There was no significant difference in the PROM scores after treatment no matter whether patients had been initially treated by an orthopaedic surgeon or by a doctor without a specialisation in orthopaedics. This indicates that the non-orthopaedic physicians treat the fracture patients with the same quality as orthopaedic specialists. However, due to the small number of patients in the two groups, the results must be interpreted with care.

### Operative versus non-operative fracture treatment

One in five of the patients needed surgery for their fractures, mostly among those with ankle fractures (1 in 3). The patients in our study had received their primary fracture treatment in the form of closed reduction and stabilizing plaster or bandage before either transport to hospital or elective control after 7–12 days.

Surgical treatment of moderately displaced distal radius fractures, especially plating procedures, have become more common during the last 20 years, as well as percutaneous pinning of unstable fractures [3, 5]. However, several studies indicate that most patients with distal radius fractures with little or no displacement can be treated conservatively. Systematic reviews and meta-analysis [3] comparing surgical with non-surgical treatment do not find that surgical treatment offers any clear clinical benefit in elderly patients with moderately displaced distal radius fractures. Conservative treatment – including a reduction of the displacement, a stabilising cast, and follow-ups to control the maintanance of the fracture reduction – still has an important place in the treatment of non-displaced and slightly displaced distal radius fractures [18]. In younger adults, however, volar plating procedures are performed on fewer fracture displacements than previously.

Conservative treatment is also suggested for most patients with collarbone fractures.

A metanalysis of internal fixation versus nonsurgical treatment in displaced midshaft collarbone fractures [4] concluded that there is lack of evidence to recommend open reduction and internal fixation (ORIF) for all displaced midshaft clavicle fractures. Comparing the long-term functional outcomes after conservative treatment versus ORIF showed no significant difference in DASH scores.

In our study, 2 of 3 of the ankle fracture patients were treated conservatively, and their FAOS scores were similar to those after treatment in hospital [4, 7, 19]. Operative treatment is of vital importance for the outcome after displaced ankle injuries. However, according to a recent meta-analysis [8], the most common non-displaced ankle fractures can be treated both surgically and conservatively with similar results.

### PROM data after treatment of the different fracture types

We have not found any PROM study from general practice in the literature. As a basis for comparison, we have used studies from hospital population. In addition, a study conducted in Norway on normative values in the general population in 2014 provides in our opinion a good base for comparison related to function after wrist and collarbone fractures. This study shows Quick Dash scores for 10-year groups from 20 years. [16]. The Quick Dash score for 20–29 years in this study was 5 for men, 6 for women. These PROM results in the range of our overall results for wrist and collarbone fractures.

The interpretation of PROM data such as Quick DASH and FAOS should be done with caution. Although data are valid and reliable, they must be viewed in relation to the clinical situation. The minimal clinically important differences for Quick DASH have been shown to be about 16, for FAOS about 14. [20, 21]

### Wrist

The mean Quick DASH score among the patients with distal radius fracture was 5.1, and the score was significantly lower for male patients than for female patients. In a meta-analysis from 2015 (3), the authors compared operative versus nonoperative treatment of distal radius fracture. The mean DASH score in papers included in the metanalysis study varied from 6.2 to 20.3, and there were no or only exceedingly small differences between the operative group and the conservatively treated group [5, 6]. Our results are somewhat better compared to this. However, we had a higher proportion of non-operated wrist fractures than in the meta-analysis, which might imply that the fractures in our study were less severe than in the hospital studies.

### Collarbone

The Quick DASH score among patients with collarbone fracture was quite low and was lower than the general Norwegian population [16]. Among the six female patients, the Quick DASH score was 0, meaning no disabilities at all. The maximum Quick DASH score in the male group was 3.0 in the age group 0–19 years. The mean Quick DASH score was 4.5 in a hospital study of functional outcome after non-operative treatment of displaced, shortened, midshaft clavicle fractures in adolescents [7]. This is equal to the normative value for the general population in Norway [16].

In our study, the mean score for the whole group of men was 2.9 (age 0–69). The mean Quick DASH score of the general population was higher, and was 5.0 for men in the vage group 20–29 years. Most collarbone fractures occur in the youngest age group. Other studies have also found low disability scores in clavicle fractures, especially in younger patients, after treatment and follow-ups in hospitals and clinics [7, 22, 23]. The even lower score in our study might be explained by the selection of younger people and well trained skiers.

### Ankle

The FAOS score for ankle fractures in our study was quite high compared to other studies, indicating good outcomes [8]. About 30% of the patients with ankle fractures in our study were referred to surgical treatment. The percentage of patients in need of operative treatment corresponds well with reports from other studies of patients primarily treated in hospitals [8, 9]. This supports our assumption that the assessments made at the primary healthcare level are able to properly select the correct patients in need of surgery. Good service and the availability of digital communication and interactions with radiological and orthopaedic services at the regional hospitals are important factors for the good results. The setting at Bykle primary healthcare centre seems to be favourable in this respect. The healthcare providers have for many years collaborated with the local hospital and have established routines regarding radiological and orthopaedic consultation. This might also be part of the reason why non-orthopaedic doctors did just as well as orthopaedic surgeons in our study.

### QOL

The QOL scores for the three types of fractures were high, more than 90, and were lowest for ankle fracture and highest for collarbone fractures. The QOL scores among the patients in our study seem to be at the same level or better compared with other studies [8].

### Satisfaction with the treatment given at the emergency primary healthcare centre

The disability scores among the patients in our study seem to be low compared to other studies. Nearly 90% of the patients were satisfied or very satisfied with the treatment they received at the primary healthcare centre. Only 1 in 10 reported that they to some, high, or very high degree had not received proper treatment. The high satisfaction with the local health service corresponds well with other studies from general practice. In a study from the Netherlands, about 80% of the patients who attended the general practitioner cooperative for consultation or those receiving a home visit reported being satisfied [24]. The study from the Netherlands focused on general consultations in primary care, not specifically fracture treatment, and the reported high degree of satisfaction with the local treatment corresponds well with the good PROM reports on function after treatment [11]. Other studies that specifically deal with treatment of fractures, both in hospitals and the primary health care service, find similar figures of patient satisfaction with diagnosing and treatment of fractures. [25, 26]

### Strengths and limitations

The survey response rate was quite good. However, we found that females had a higher response rate than males. This might have caused a bias in our study, but a similar gender difference in response rate is also found in other studies [27].

The analysed material is relatively old. Possible change in clinical practice due to the increasing recruitment problems in general practice in Norway the last 10 years could have influenced the approach to diagnostics and treatment of fractures. However, the situation in this municipality is unchanged.

A strength of this study is that the starting point for selecting patients was a diagnosed fracture by a radiologist or orthopaedic surgeon verified in the medical records for each patient. The data from patients’ medical records and questionnaires were thoroughly registered and analysed by the first author, who himself is a clinician with extensive knowledge of the Bykle primary healthcare centre.

A limitation is the relatively small patient groups in the subgroups of fractures, especially ankle fractures. This makes it difficult to obtain significant results of possible differences in outcome.

The sample of fracture patients represents a quite young population, often active skiers and other winter sport athletes. This is a limitation for generalizing the findings to populations that include older patients.

Another limitation is the sample period that lasted for 5 years. The time from actual injury to answering the questionnaire varied from 9 months to 5 years and 9 months. Remembering details after such a long time may result in recall bias [28], inducing uncertainty that especially applies to the questions concerning matters at the time of the injury, such as questions about satisfaction with the treatment. However, the PROMs asking about function and pain in the last weeks before the patient was filling out the questionnaire would not be affected by possible recall bias.

Few studies have analysed children’s ability to answer a Quick DASH questionnaire, but modified versions are developed for children [29]. In our study, we chose to ask parents to help their children to answer and to include these results in our study. We believe that these answers are as valuable as the self-reported PROM data from the adult population.

The extent to which the results from this study can be generalized to other municipalities or populations is open for discussion. The survey was conducted at a ski destination with a predominance of young active people, and it gives an increased fracture frequency and also a skewed distribution of injuries compared to the average in a general population. The personnel working in the study municipality is specifically trained and receive an update in X-ray assessment and conservative fracture treatment, but this training is limited. However, also elsewhere in Norway and Europe x-ray examinations and fracture treatment are carried out and X-rays are sent to the nearest hospital for assessment and can be discussed with a radiologist and orthopedist. [25]

## Conclusion

The patients reported low rates of functional disability and high rates of satisfaction after initial radiological diagnosis and treatment of their fracture. PROMs among collarbone and ankle fracture patients showed no clinically relevant age or gender differences. Specialisation of treating physician did not influence our results in any of the fracture types. The general satisfaction with the treatment given was good for all fracture types in our study.

### Implications

Based on the high degree of patient-reported satisfaction with the local emergency primary healthcare service and the good outcome reports from treated fracture patients, we find it safe to recommend conservative fracture treatment in primary healthcare. However, this requires binding, concomitant, and secure decision support from orthopaedic surgeons and radiologists, digital radiological equipment, and continuous and updated local education and training in diagnostics and treatment.

With an increasing number of older and less mobile patients, especially in rural districts, we think it is time to move a larger part of the conservative fracture treatment from hospitals to primary healthcare. However, this will need more research to ensure that patients receive good evidence-based treatment in the municipality that is not inferior to the outcome and satisfaction after hospital treatment.

There is need for more studies comparing functional outcome of conservative fracture treatment in primary care versus hospital. [30]

## Data Availability

The datasets used and/or analysed during the current study are available from the corresponding author on reasonable request.

## Abbreviations

PROM: Patient Reported Outcome Measure
DASH: Disability of Arm, Shoulder and Hand
FAOS: Foot and Ankle Outcome Score
ORIF: Open reduction and internal fixation
